# Digital Implant-Supported Restoration Planning Placed in Autologous Graft Using Titanium Implants Produced by Additive Manufacturing

**DOI:** 10.3390/dj12070192

**Published:** 2024-06-24

**Authors:** Rafael Seabra Louro, Vittorio Moraschini, Fernando Melhem-Elias, George Patrick Sotero Sturzinger, Renata Augusto Amad, Jamil A. Shibli

**Affiliations:** 1 Department of Oral Surgery, School of Dentistry, Fluminense Federal University, Niterói 24020-140, Brazil; rafaelseabra@id.uff.br (R.S.L.); vitt.mf@gmail.com (V.M.); g.sotero1995@gmail.com (G.P.S.S.); 2 Department of Oral and Maxillofacial Surgery, School of Dentistry of the University of São Paulo, São Paulo 05508-000, Brazil; fmelias@usp.br; 3 Department of Periodontology, Dental Research Division, Guarulhos University, Guarulhos 07023-040, Brazil; renataaugusto@hotmail.com

**Keywords:** mandible reconstruction, autologous graft, dental implants, virtual planning, case report

## Abstract

This clinical report presents a technique to reconstruct extensively resected mandibles using a combination of autologous bone grafts and additive manufacturing techniques. Mandibular defects, often arising from trauma, tumors, or congenital anomalies, can severely impact both function and aesthetics. Conventional reconstruction methods have their limitations, often resulting in suboptimal outcomes. In these reports, we detail clinical cases where patients with different mandibular defects underwent reconstructive surgery. In each instance, autologous grafts were harvested to ensure the restoration of native bone tissue, while advanced virtual planning techniques were employed for precise graft design and dental implant placement. The patients experienced substantial improvements in masticatory function, speech, and facial aesthetics. Utilizing autologous grafts minimized the risk of rejection and complications associated with foreign materials. The integration of virtual planning precision allowed customized solutions, reducing surgical duration and optimizing implant positioning. These 2 cases underscores the potential of combining autologous grafts with virtual planning precision and dental implants produced by additive manufacturing for mandible reconstruction.

## 1. Introduction

Mandible resections, often necessitated by trauma, tumors, or congenital deformities, present intricate challenges due to their vital role in facial aesthetics and function [1]. The subsequent reconstruction process frequently relies on autologous grafts, which offer optimal biocompatibility and minimized rejection risks [2].

Reconstruction is imperative to restore both form and function [3]. Autologous grafts, vascularized or not, harvested from the patient’s bone, exhibit superior osteogenic potential and reduce immunogenic complications. Common graft sources include the iliac crest, fibula, and scapula, each offering distinct advantages depending on the extent of resection technological advancements, such as three-dimensional (3D) printing and computer-assisted virtual planning, enhanced graft precision, and expedited surgical planning [4].

Rehabilitation following reconstruction often involves dental implants, reinstating oral function, and facial symmetry. Dental implants provide stability for prosthetic restorations, ensuring proper occlusion and preventing bone resorption [5]. However, successful implantation hinges on adequate bone volume and vascularity, highlighting the significance of meticulous graft placement. Osseointegration is influenced by graft quality, surgical technique, and implant surface topography. Recently, the use of 3D-printed dental implants, or more specifically, additive-manufacturing titanium dental implants, have been produced to offer a higher area of surface topography to facilitate the very early stage of protein adsorption [6], and consequently increase the bone-to-implant contact [7,8].

Formerly known as direct metal laser sintering (DMLS) or direct laser metal forming [7], the implants produced by additive manufacturing printed out the core of the implant using titanium grade 23 using a power laser that melts each titanium particle, providing a unique surface [8,9,10] with a high roughness process that presented a higher contra-torque retrieval and higher bone-to-implant contact. These processes play a pivotal role in the bone behavior around the implants and could improve 2-fold better osseointegration when compared with standard surfaces [6,7,8].

Comprehensive rehabilitation demands a multidisciplinary approach involving oral and maxillofacial surgeons, plastic surgeons, prosthodontists, and physical therapists. Preoperative evaluation, precise surgical execution, and prosthetic rehabilitation constitute a continuum of care essential for favorable outcomes.

Mandible resections prompt the need for meticulous reconstruction and subsequent rehabilitation. Autologous grafts serve as invaluable tools in achieving functional and aesthetic restoration [3,9]. The integration of dental implants further augments oral rehabilitation. A nuanced understanding of the intricate relationship between mandibular anatomy, autologous grafts, and dental implants is pivotal for optimizing outcomes and enhancing the lives of patients undergoing these procedures.

The 2 cases described in this article were rehabilitated with dental implants produced by printing (additive manufacturing). The additive manufacturing technique for implants presents some advantages such as improving mechanical properties, reducing adhesion and microbial colonization, and increasing the adhesion of the fibrin network [6,10].

Few studies report the rehabilitation of extensive bone defects through virtual planning. In this way, this report aimed to show the benefits of the integration of autografts and dental implant rehabilitation, using surgical guided by virtual planning precision; showcasing promising advancements in mandible reconstruction.

## 2. Materials and Methods

### 2.1. Case 1

**Figure 1:**
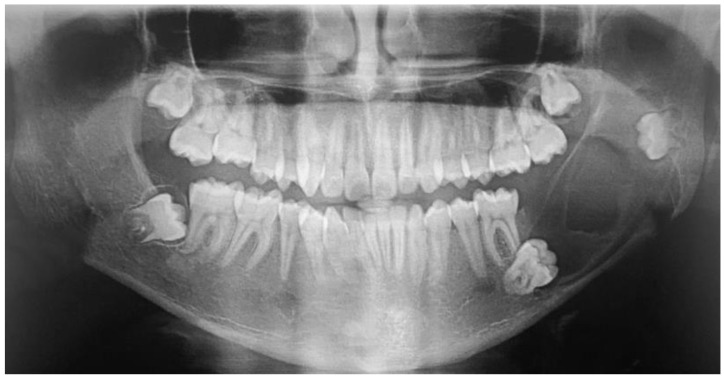
Preoperative panoramic.

**Figure 2:**
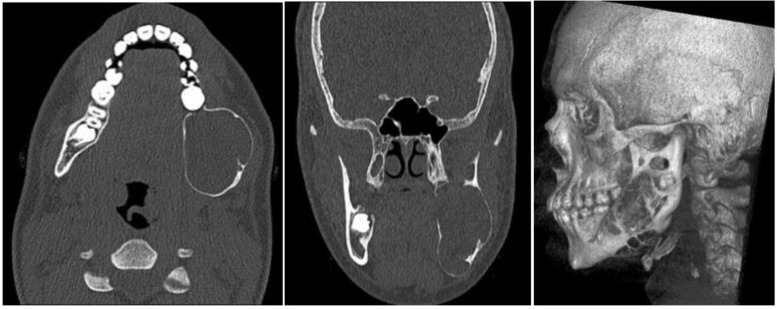
Computed tomography.

A 17-year-old male presented with a painless swelling in the left mandible. After a thorough clinical and radiographic evaluation and incisional biopsy, the diagnosis of Ameloblastoma was made. His clinical examination revealed a severe extension in the left posterior mandible (Figure 1 and Figure 2).

**Figure 3:**
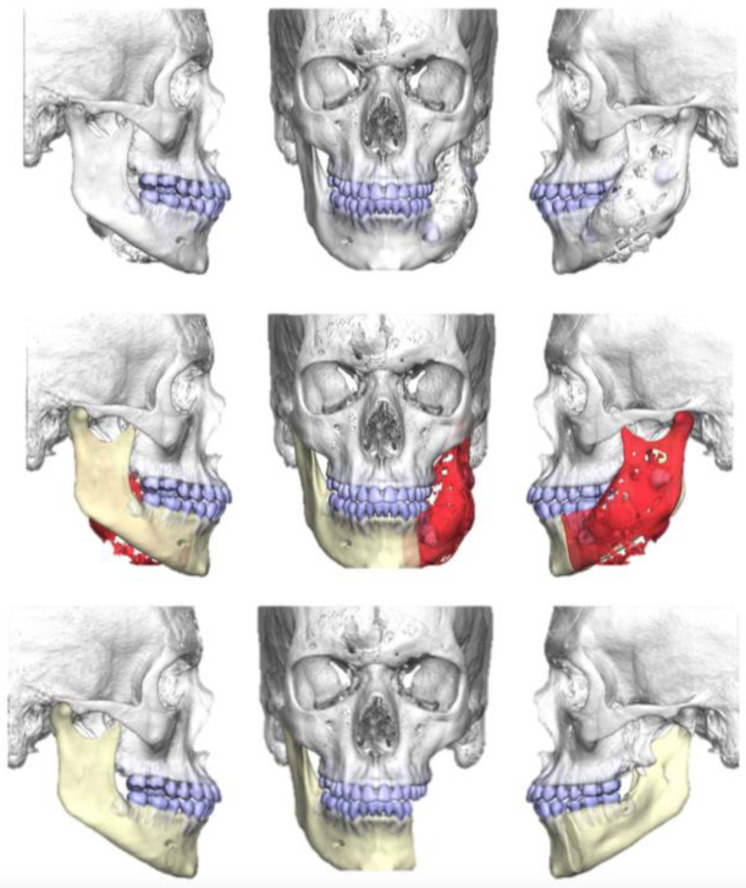
Digital planning of the tumor resection area.

**Figure 4:**
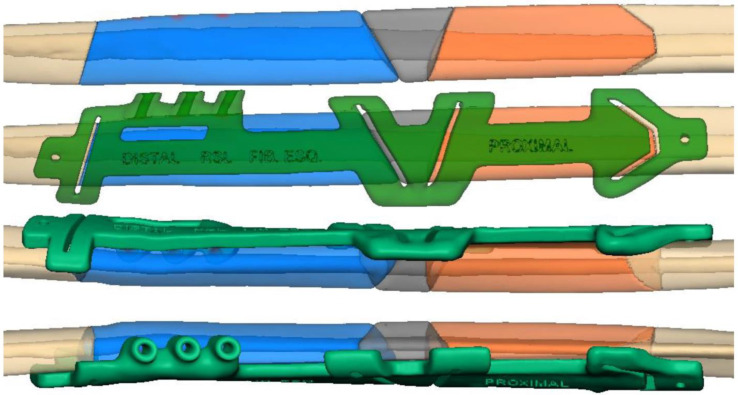
Planning the fibula cutting guide according to the dimensions of the mandible reconstruction area.

**Figure 5:**
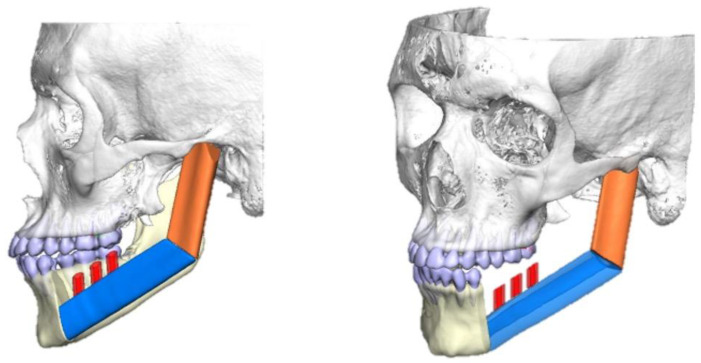
Reconstruction planning with guides for immediate dental implant placement.

**Figure 6:**
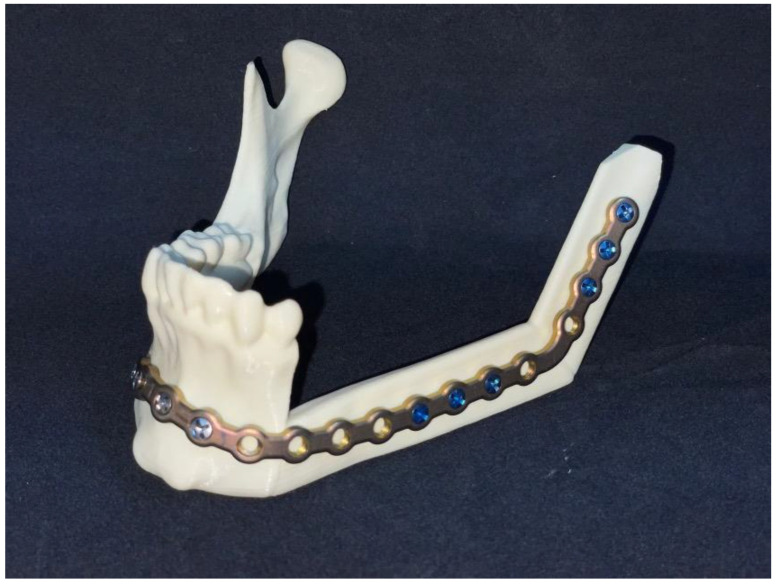
3D printed model.

**Figure 7:**
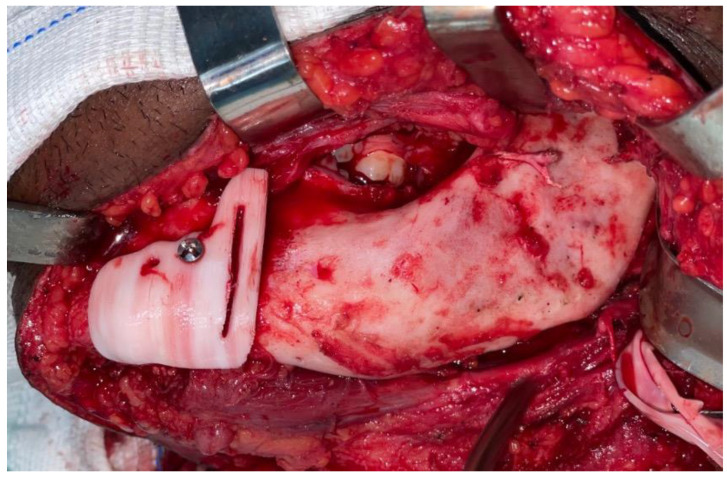
Transoperative mandible resection.

**Figure 8:**
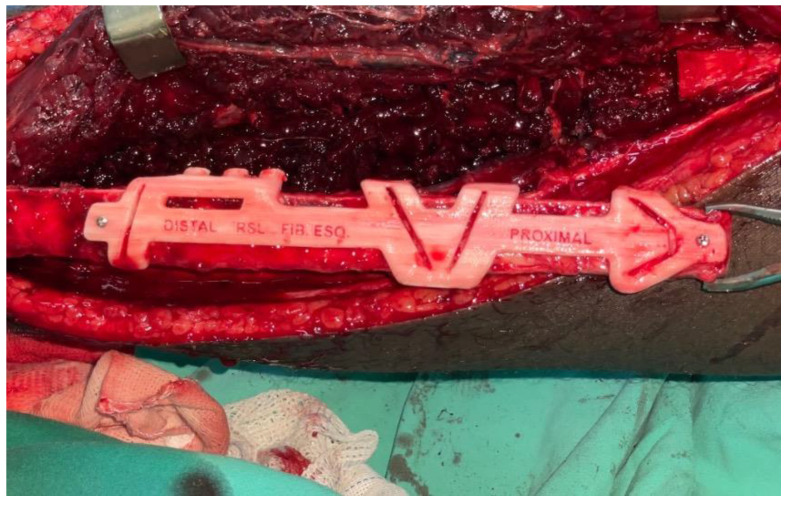
Transoperative fibula resection.

**Figure 9:**
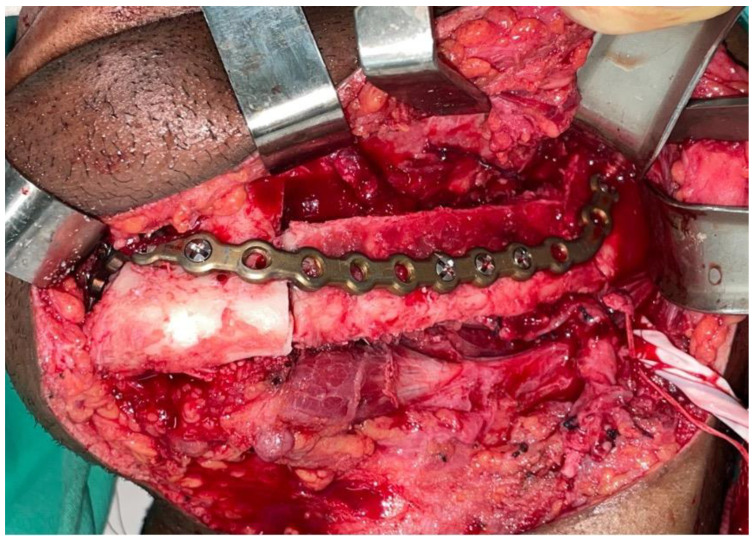
Transoperative fibula flap positioned.

The resection of the lesion and removal of the graft from the donor area were performed using digital surgical planning (Mimics, Materialise, Leuven, Belgium). The data for virtual planning was conducted from previous cone-beam computed tomography (CBCT) (3D Accuitomo, Morita, Tokyo, Japan) (DICOM file) performed in the donor area (fibula) and CBCT plus intraoral scanning (Virtuo Vivo, Straumann, Curitiba, Brazil) in the recipient area (mandible). Digital planning can be seen in Figure 3, Figure 4 and Figure 5. Surgical resection of the tumor was performed, followed by immediate reconstruction using a microvascularized fibula flap graft. The virtual planning of the resection and reconstruction allowed the mandible contouring, reducing the surgical duration and optimizing the positioning of the graft (Figure 6, Figure 7, Figure 8 and Figure 9).

**Figure 10:**
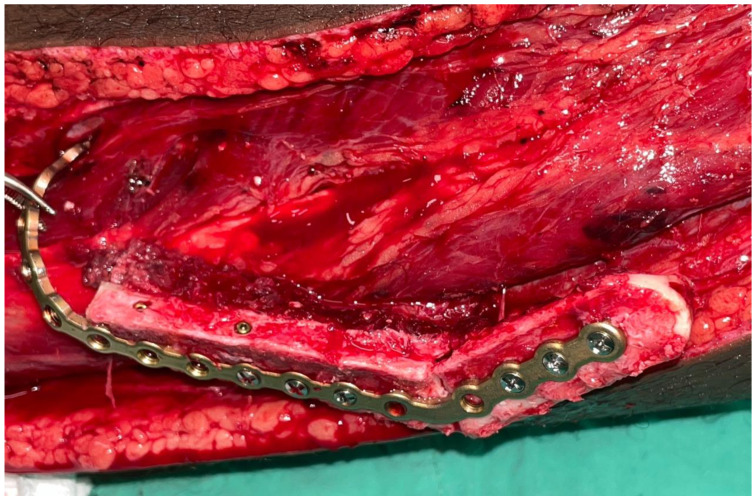
Dental implants placed.

**Figure 11:**
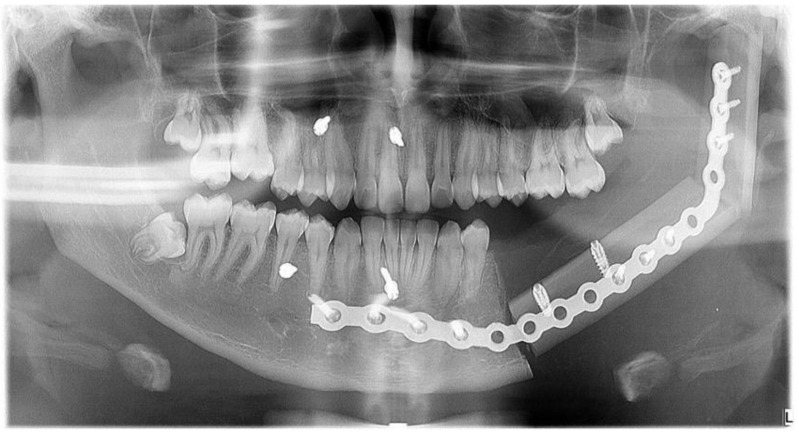
Panoramic radiographic, immediate postoperative.

**Figure 12:**
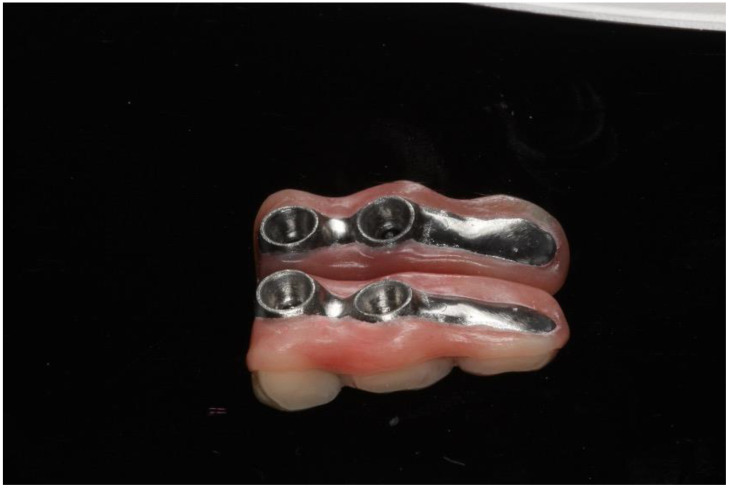
Final prosthesis.

**Figure 13:**
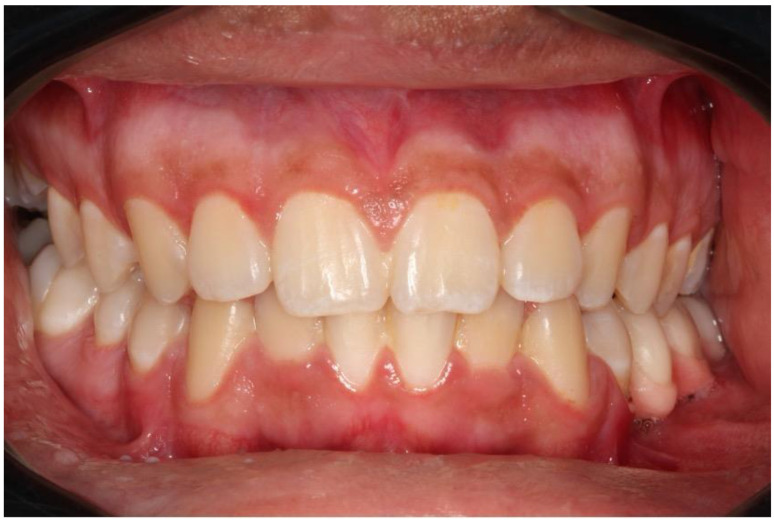
Prosthetic rehabilitation of case 1.

Immediate additive manufacturing titanium dental implant placement (4.0 × 11 mm RE, Plenum Bioengenharia, Jundiaí, Brazil) was attempted in this case, and both were well positioned (Figure 10 and Figure 11). The positioning of the implants was based on the remaining teeth and the previously performed virtual diagnostic wax-up. After a healing period of 4 months, the patient underwent successful rehabilitation with fixed implant-supported prostheses, restoring function and aesthetics (Figure 12 and Figure 13).

### 2.2. Case 2

**Figure 14:**
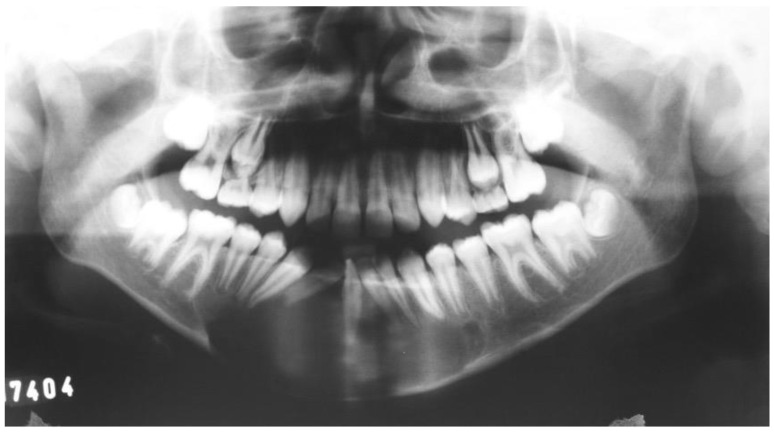
Initial ameloblastoma.

**Figure 15:**
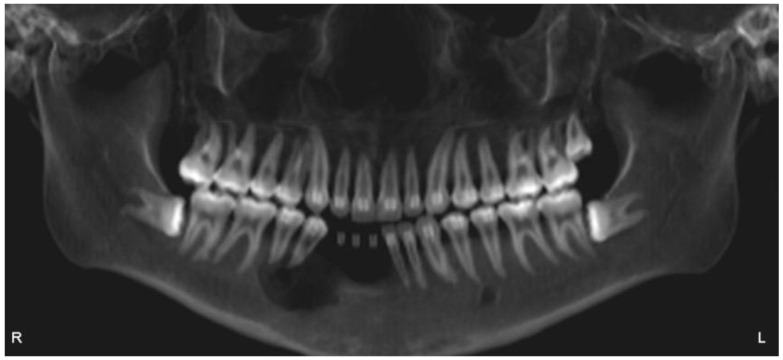
Recurrent ameloblastoma.

**Figure 16:**
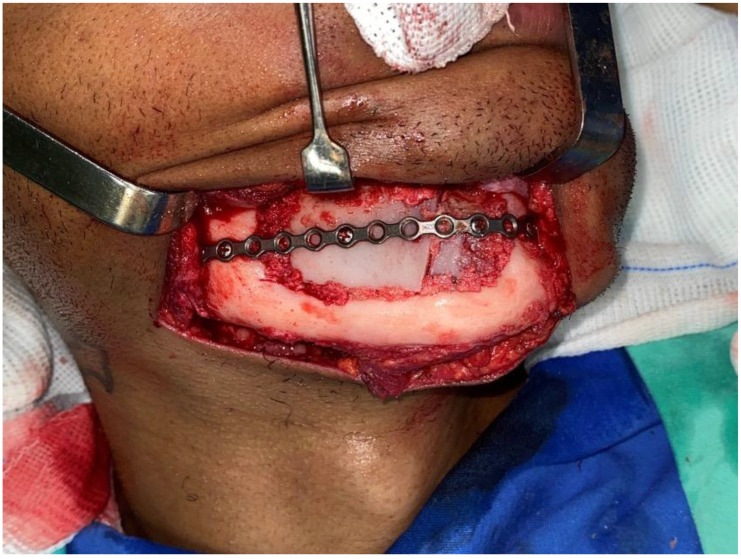
Mandibular reconstruction with iliac crest.

**Figure 17:**
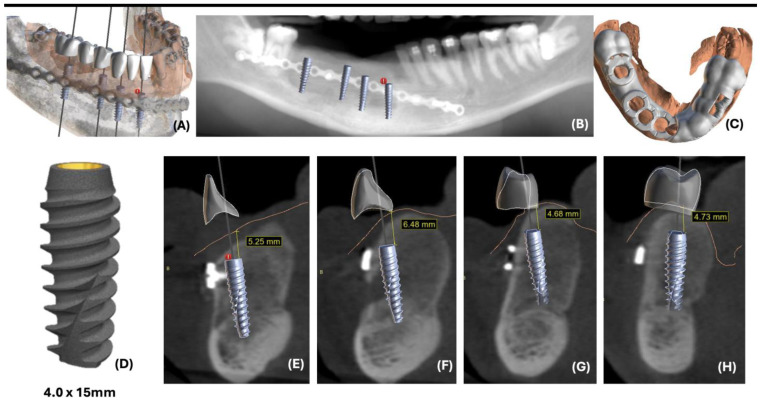
(**A**) Digital planning showing the implants oriented prosthetically; (**B**) Radiographic view of the planned implants; (**C**) surgical guide; (**D**) dental implant; (**E**–**H**) sagittal view of the implants placed in positions 41, 43, 44, and 46 respectively.

**Figure 18:**
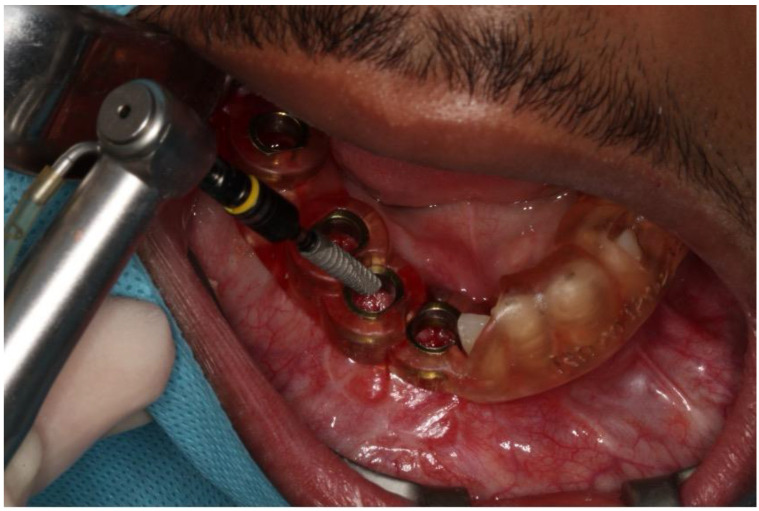
Guided implant installation.

**Figure 19:**
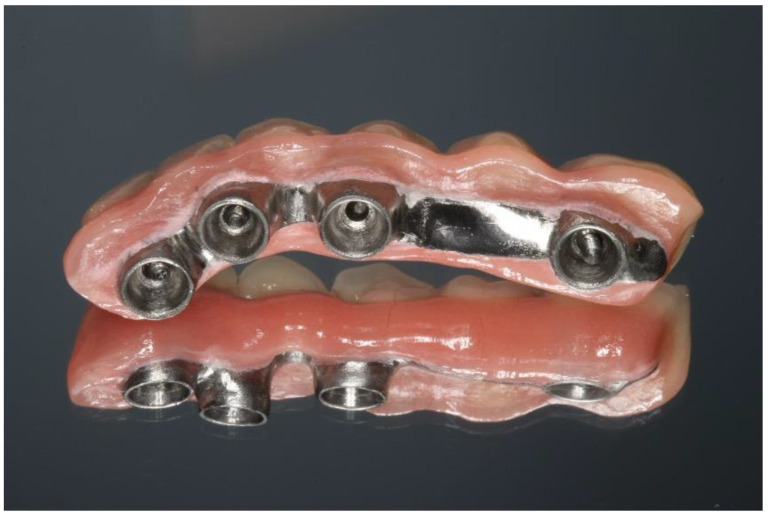
Dental prosthesis.

**Figure 20:**
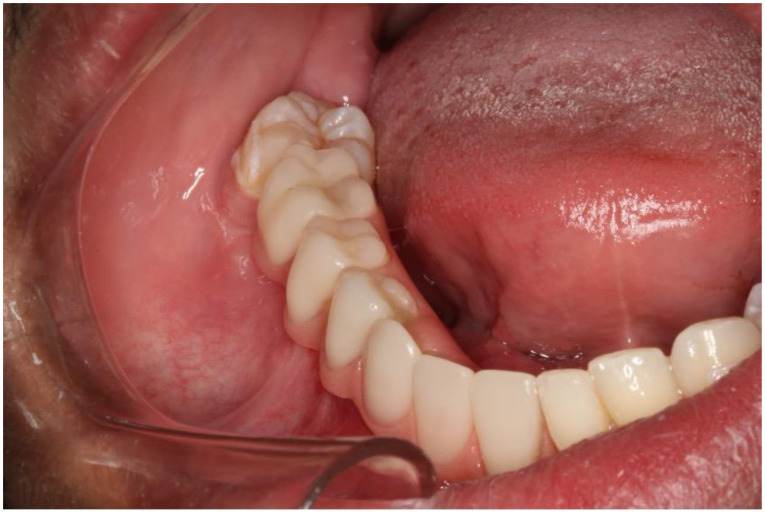
Prosthetic rehabilitation.

**Figure 21:**
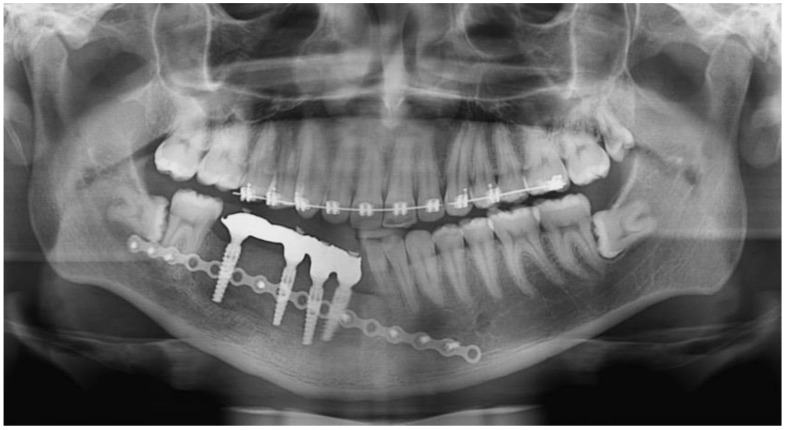
Panoramic radiographic view.

This case report presents the management of recurrent ameloblastoma occurring 12 years after initial treatment (Figure 14). A 21-year-old male patient presented with a recurrent lesion in the anterior mandibular region (Figure 15). A thorough clinical and radiographic examination confirmed the diagnosis of a recurrent ameloblastoma. Surgical resection of the lesion was performed, involving a wide margin to ensure complete removal, preserving the mandibular basal bone in the anterior region of the mandible. Subsequently, after 2 years, a reconstruction procedure was carried out utilizing an iliac crest graft to restore the mandibular continuity (Figure 16). Following six months of the healing period, four dental implants (Plenum, RE, Jundiaí, Brasil) were successfully placed in the reconstructed area. Virtual planning (software BlueSky Plan version 4.12, Libertyville, IL, USA) was used to fit the implants in a better position (Figure 17 and Figure 18). The patient achieved functional and aesthetic rehabilitation with a fixed implant-supported prosthesis (Figure 19, Figure 20 and Figure 21).

## 3. Discussion

One of the most remarkable advancements in mandible reconstruction is the use of autografts. Autografts involve transferring bone tissue from the patient’s body to the mandible site that requires reconstruction. Autografts can be of intraoral (e.g., mandibular ramus and para symphysis) or extraoral origin, depending on the amount of graft needed. This technique offers several advantages over synthetic materials or allografts. Autografts have a lower risk of rejection, infection, or other complications because the body recognizes and accepts its tissue [11]. Using the patient’s bone also ensures a perfect fit in terms of shape and size, which is crucial for functional and aesthetic success. In addition, the virtual planning using digital tools to the reported patients improved the clinical outcome and reduced the morbidity of the patients.

The natural contours and appearance of the patient’s face are preserved, improving the aesthetic outcomes of the procedure, reducing the risk of disfigurement, and enhancing the patient’s self-esteem. This approach is essential for patients who have undergone traumatic accidents or cancer surgeries, as it helps them regain a sense of normalcy in their appearance.

In addition to aesthetic benefits, autologous grafts offer enhanced functional restoration. The patient’s bone provides the necessary structural support and strength for proper chewing and speaking [11,12]. This ensures that the patient can return to a relatively normal life with minimal hindrance in their daily activities. Improved functionality enhances the patient’s quality of life and reduces the need for additional surgeries or adjustments [13,14].

Dental implant rehabilitation is another pivotal aspect of mandible reconstruction [15]. Once the bone structure has been restored using autologous grafts or other techniques, the next step is to rehabilitate the patient’s oral function. For patients who have an indication, dental implants are the gold standard for replacing missing teeth [16], and they play a vital role in mandible reconstruction. These implants are surgically placed into the restored bone, anchoring prosthetic teeth. It allows for the restoration of oral functionality in a way that closely mimics natural teeth. Unlike traditional dentures, dental implants are firmly anchored in the jawbone, providing stability and preventing issues related to loose or bad-fitting dentures. Patients who have undergone mandible reconstruction with dental implants can regain the ability to eat a wide variety of foods and speak with confidence [17].

Using dental implants produced by additive manufacturing in mandible reconstruction significantly enhances the osseointegration process [7,8] and the patient’s quality of life [15]. The 3D printing process using the micro-fusion of titanium particles provided a surface that enhances the bone anchorage due to the rough surface [8] increasing the mechanical stability. However, these implants present a morse tapper connection prepared by milling machined as the additive manufacturing process did not allow the production of the connection part between the implant and abutment. Patients no longer have to deal with the inconveniences and discomfort associated with removable dentures. They can enjoy a varied diet and social interactions without fearing their dentures slipping or causing embarrassment. This improvement in the patient’s daily life cannot be understated.

Another important characteristic of the implants produced by additive manufacturing is the final implant surface topography. This surface depicted a rough surface with Ra = 10.82 um that improves the bone cells and proteins adhesion as previously described [7]. Meantime, there are some concerns about microbial adhesion and further peri-implant bone loss due to the great roughness. A vitro study [10] evaluated the adhesion of a polymicrobial biofilm formed by 31 bacterial species often found around peri-implantitis and periodontitis. The authors showed that the 3D printed surface did not increase the number of microorganism adhesion when compared with as-machined and sandblasted acid-etched surfaces. This data could suggest that the grater Ra present in the additive manufactured surfaces should not be a problem for long-term follow-up of these implants.

Virtual planning precision is a recent technological advancement that has revolutionized the field of mandible reconstruction [5,18]. By utilizing advanced imaging techniques such as CT scans and 3D modeling, surgeons can now plan the entire procedure in a virtual environment before the actual surgery. This level of precision offers numerous benefits such as reduced time-consuming procedures, 3D implant placement, better implant-supported restoration delivery, and finally, best patient outcome [19].

With virtual planning precision, surgeons can meticulously plan the reconstruction procedure, ensuring that every step is carried out with the utmost accuracy. This reduces the risk of surgical complications and allows for a smoother, faster, and less invasive procedure [20]. Surgeons can identify potential challenges and address them before they become significant issues. It allows a customized treatment plan for each patient. Surgeons can tailor the reconstruction to the patient’s specific needs and anatomical characteristics. This individualized approach results in a more precise and successful outcome, whether using autologous grafts, dental implants, or a combination of techniques [21].

Due to the accuracy and precision of virtual planning, the surgery itself is often shorter, and the recovery period is more predictable. Patients can typically resume their regular activities sooner, and the risk of post-operative complications is reduced [22,23,24]. The patients related to the cases described are followed longitudinally within a maintenance program. Monitoring involves periodic clinical and radiographic examinations (every 6 months).

Finally, the data obtained from these 2 case reports should be considered with caution, and future studies with large samples and long-term follow-up must be done.

## 4. Conclusions

In conclusion, the combination of extraoral autografts with dental implants produced by additive manufacturing processes placed through virtual planning precision has ushered in a new era of mandible reconstruction. These advancements not only enhance aesthetic outcomes but also improve functional restoration and overall quality of life for patients when dental implants with a surface topography allow a better biomechanical anchorage. Further studies must evaluate the longitudinal behavior of these implant-supported restorations.

## Data Availability

No new data were created or analyzed in this study. Data sharing does not apply to this article.
